# Performance Validation of ORTHOSEG, a Novel Artificial Intelligence Tool for the Segmentation of Orthopantomographs and Intra-Oral X-Rays

**DOI:** 10.3390/clinpract16030054

**Published:** 2026-03-04

**Authors:** Giuseppe Cota, Gaetano Scaramozzino, Marco Chiesa, Lelio Gennaro, Maurizio Pascadopoli, Andrea Scribante, Marco Colombo

**Affiliations:** 1 Scaramozzino Dental Practice, 45020 Villanova del Ghebbo, RO, Italy; giuseta@gmail.com (G.C.); gaetano.scara87@gmail.com (G.S.); 2 Section of Dentistry, Department of Clinical, Surgical, Diagnostic and Pediatric Sciences, University of Pavia, 27100 Pavia, PV, Italy; marco.chiesa@unipv.it (M.C.); leliogennaro@gmail.com (L.G.);

**Keywords:** dental radiography, orthopantomography, periapical radiography, bitewing radiography, deep learning, convolutional neural network, image segmentation, automated segmentation, computer-aided diagnosis

## Abstract

Background: Dental radiographs are essential for diagnosis and treatment planning in modern dentistry. However, their manual interpretation is time-consuming and subject to variability, highlighting the need for automated tools to improve efficiency and consistency. This study aims to validate ORTHOSEG, a deep learning-based system designed to automate the segmentation of anatomical, pathological, and non-pathological elements in radiographs, including orthopantomograms, bitewings, and periapical images. Methods: ORTHOSEG’s performance was evaluated using a rigorously curated dataset of 150 dental radiographs, including 50 orthopantomograms, 50 bitewings, and 50 periapical images, with manual annotations by expert clinicians serving as the ground truth. The system’s segmentation performance was assessed using standard evaluation metrics, including mean Dice Similarity Coefficient (*mDSC*) and mean Intersection over Union (*mIoU*), and inference time was also recorded. Results: The system achieved high accuracy, with *mDSC* and *mIoU* values of 0.635 ± 0.233 and 0.576 ± 0.214, respectively. In particular for orthopantomograms, it achieved an *mDSC* of 0.756 ± 0.174 and an *mIoU* of 0.684 ± 0.172, surpassing existing benchmarks. Its segmentation capabilities extend to approximately 70 distinct elements, underscoring its comprehensive utility. The system demonstrated efficient computational performance, with processing times of 19.745 ± 3.625 s for orthopantomograms, 8.467 ± 0.903 s for bitewings, and 5.653 ± 0.897 s for periapical radiographs on standard clinical hardware. Conclusions: ORTHOSEG demonstrates efficiency suitable for integration into routine workflows. This study confirms ORTHOSEG’s reliability and potential to improve diagnostic workflows, offering clinicians a valuable tool for faster and more detailed radiograph analysis. Future research will focus on extending validation across diverse clinical scenarios to ensure broader applicability. However, this study has limitations, including the use of a dataset derived from a European population and the absence of usability and clinical workflow evaluation, which should be addressed in future studies.

## 1. Introduction

Dental radiographs are indispensable tools in modern dentistry, providing clinicians with critical insights into the anatomical structures and pathological conditions of the oral and maxillofacial regions [1]. They play a key role in diagnosing, planning treatments, and monitoring therapeutic outcomes, especially in managing conditions such as caries, periodontal disease, and bone resorption. Among the most commonly employed radiographic modalities are orthopantomograms, bitewings, and periapical images, which collectively enable comprehensive evaluation of dental hard tissues and disease states [2]. Despite their importance, interpreting radiographs is a time-consuming and cognitively demanding task. Manual interpretation requires significant focus and precision, leaving clinicians vulnerable to errors arising from fatigue or lapses in concentration, particularly in high-volume settings. These challenges can delay the diagnostic process and, in some cases, compromise accuracy, underlining the need for tools that can enhance efficiency without sacrificing reliability [3]. Advances in artificial intelligence (AI) have introduced automated segmentation systems capable of identifying and delineating structures in radiographic images [4,5,6]. Early methods based on traditional computer vision techniques, such as thresholding and region-growing algorithms, demonstrated some utility but struggled to handle variability in image quality and patient anatomy [7]. Deep learning has since emerged as a transformative approach, leveraging Convolutional Neural Networks (CNNs) to address these limitations. By learning features directly from data, CNN-based models have achieved significant success in segmentation tasks, such as caries detection [8,9,10,11], bone loss assessment [12,13,14], and anatomical labeling [15,16,17]. ORTHOSEG (ORTHOpanoramic and intraoral SEGmentation) is a deep learning-based AI system integrated into the Neowise software (version 1.5, CEFLA S.C., Imola, Italy). The core of the system consists of a suite of specialized deep learning models belonging to the convolutional neural network (CNN) family, with each model dedicated to a specific predictive task. In particular, these models are based on the Mask R-CNN (Region-based Convolutional Neural Network) architecture, which enables instance segmentation by performing pixel-level detection and classification of individual anatomical and pathological structures within radiographic images [18]. It offers automated segmentation of anatomical, pathological, and non-pathological elements in radiographs. Designed to improve diagnostic workflows, ORTHOSEG (CEFLA S.C., Imola, Italy) classifies each pixel to identify and delineate structures across various radiographic modalities, including orthopantomograms, bitewings, and periapicals. Its primary goal is to assist clinicians by providing a rapid and detailed visualization of radiographic findings, allowing them to focus on interpretation and decision-making while reducing the cognitive load associated with manual analysis. This study aims to validate the performance of ORTHOSEG (CEFLA S.C., Imola, Italy), assessing its accuracy and reliability in segmenting anatomical, pathological, and non-pathological elements in dental radiographs. The evaluation is based on comparing the system’s output against manual segmentations performed by experienced clinicians, which serve as the ground truth. By assessing its accuracy and reliability across different radiographic types, this research seeks to determine whether ORTHOSEG (CEFLA S.C., Imola, Italy) meets the standards required for integration into routine dental workflows. The research hypothesis of this study is that ORTHOSEG can achieve segmentation performance with quantitative accuracy metrics sufficient to enable reliable identification of anatomical, pathological, and non-pathological structures in orthopantomograms, bitewings, and periapical radiographs, supporting its applicability in routine clinical practice.

## 2. Materials and Methods

ORTHOSEG (ORTHOpanoramic and intraoral SEGmentation) is a deep learning-based AI system integrated into the Neowise software (CEFLA S.C., Imola, Italy). Table 1 reports the elements detected by the ORTHOSEG system. The materials and methods employed to assess and validate the performance of ORTHOSEG are illustrated, with the aim of demonstrating its potential clinical utility.

### 2.1. Dataset

A diverse cohort of 50 orthopanoramics, 50 bitewings and 50 periapicals was selected using different devices and varying acquisition parameters. Patients ranged from 8 to over 65 years old, representing a broad spectrum of European patients, without differentiating between ethnic groups and including both sexes. To analyze the impact of dental development, patients were categorized into two groups: mixed dentition (8–12 years) and permanent dentition (13–65 years). The distribution of these images is summarized in Table 2, Table 3 and Table 4. Specifically, the dataset comprised 50 orthopantomograms, 50 bitewings, and 50 periapical radiographs, ensuring balanced representation across radiographic modalities. For orthopantomograms, the sample included 28 male and 22 female patients, with 6 children (8–12 years), 32 adolescents and adults (13–65 years), and 12 elderly patients (>65 years). Bitewing radiographs included 25 male and 25 female patients, with 6 children, 27 adolescents and adults, and 17 elderly patients. Periapical radiographs included 22 male and 24 female patients, with 5 children, 8 adolescents and adults, and 37 elderly patients. This distribution ensured adequate representation of demographic subgroups for performance evaluation. All radiographs were anonymized before analysis and included cases with both pathological and non-pathological findings, such as agenesis, caries, lesions [19], extractions, fillings [20], root canal treatments [21], orthodontic brackets [22], and orthodontic wires. The sample size was determined based on a trade-off between quantity and quality. These images were obtained via consecutive selection to reflect a “real-world” distribution of dental anatomy, patient demographics, and pathological findings. By utilizing 50 samples per modality, the study ensured a sufficient sample size to satisfy the rule of thumb for the Central Limit Theorem (n > 30), ensuring that the evaluation metrics used are sufficiently reliable for each imaging type. Moreover, the inclusion of different age groups (children to elderly) naturally introduced a variety of developmental and degenerative conditions. The sample size was also influenced by the extremely time-consuming activity of defining the ground truth. In our labeling process, where each image is reviewed by at least two expert clinicians, we prioritized high-quality annotations over quantity to ensure a more reliable dataset. Finally, these sample sizes are comparable to those reported in the literature, often limited to one modality. For instance, in [9] the authors used 53 bitewings to test the system CranioCatch (Eskişehir, Turkey), while other studies tested commercial software such as the Diagnocat AI system (Miami, FL, USA) on 55 orthopanoramics [23], 214 periapicals, 24 bitewings [24] and 20 periapicals [25]. The radiographs were retrospectively collected from the clinical imaging archives of the Dental Clinic of the University of Pavia (Pavia, Italy). All radiographs were fully anonymized prior to analysis to ensure patient confidentiality and compliance with ethical and data protection regulations. The study was conducted in accordance with the Declaration of Helsinki and approved by the Unit Internal Review Board (approval number: 2025-1112). No formal a priori sample size calculation was performed, as this study was designed as a performance validation study of an AI system using a predefined annotated dataset rather than a prospective hypothesis-driven clinical trial. The dataset included 150 radiographs equally distributed among orthopantomograms, bitewings, and periapical images, which was considered sufficient to ensure a balanced and representative evaluation of the system’s segmentation performance across different radiographic modalities and demographic subgroups. This sample size is consistent with previously published validation studies in dental AI.

### 2.2. Ground Truth Definition for System Validation

The construction of the validation dataset was carried out to ensure high reliability and accuracy in evaluating the system’s performance. The annotation process of the validation dataset involved two experienced clinicians, each with a minimum of five years of clinical practice, who fulfilled complementary roles as annotator and reviewer. This dual-review process was designed to minimize bias and maximize the quality of the ground truth data, which serves as the reference standard for performance benchmarking. CVAT (version 2.21.0, CVAT.ai, Wilmington, DE, USA- https://github.com/cvat-ai/cvat accessed on 30^th^ January 2026), a self-hosted web application, was used as the annotation tool, allowing for the manual classification and annotation of images. Additionally, the self-hosted solution enables the installation of the annotation system on a server physically located in the European Union, ensuring full compliance with GDPR (General Data Protection Regulation). The annotation process was divided into two distinct phases [26]:

1. Initial Annotation: The annotator performed manual segmentations on each radiography using the annotation tool.
2. Review and Quality Assurance: The reviewer meticulously examined the initial annotations to ensure their accuracy and adherence to established anatomical guidelines. Using cross-referencing techniques and visual validation, the reviewer corrected any discrepancies identified in the annotations. This phase also involved quality control checks to address any issues related to image resolution, segmentation consistency, or potential operator errors.

### 2.3. System Performance Validation

The performance evaluation is conducted by comparing the automatic detections against a testing dataset that serves as the ground truth [27,28]. The testing dataset was created as described in Section 2.2. The performance validation process utilizes a range of best-practice metrics to comprehensively assess the system’s performance [29]. This validation is run on a computer with an NVIDIA RTX A2000 GPU (VRAM 12 GiB, NVIDIA Corporate, Santa Clara, CA, USA) and an Intel Core i5-13500 CPU (2.50 GHz with 16 GiB of RAM, Intel Corporation, Santa Clara, CA, USA).

#### 2.3.1. Metrics

This subsection describes the quantitative metrics adopted for the validation of ORTHOSEG. These metrics are widely adopted in the field of computer vision and medical image analysis for assessing the performance of segmentation systems [30,31]. All metrics were implemented internally in Python (version 3.8.18, Python Software Foundation, Wilmington, DE, USA).

The null hypothesis for the statistical analyses posited that no significant differences existed between the mean performance metrics (*mDSC*, *mIoU*, *mPrecision*, and *mRecall*) across the evaluated subgroups, including sex and age categories. These hypotheses were tested using a two-tailed independent *t*-test for sex-based comparisons and ANOVA for age-based comparisons, with statistical significance set at *p* < 0.05.

**Mean Intersection over Union (*IoU*).**

*IoU* is the ratio of the overlap area between the predicted object and the ground truth (correct) object to the area of their union [32]. It measures how well the predicted object overlaps with the actual object.

The *IoU* is defined as

$$IoU=\frac{Inter\sec tionArea}{UnionArea}$$

A higher *IoU* indicates better overlap, meaning the predicted object more accurately matches the ground truth.

The *mIoU* computes the average *IoU* across all categories [33]. The *mIoU* is defined as

$$mIoU=\frac{1}{C}\sum_{i=1}^{C}\frac{TPi}{TPi+FPi+FNi}$$

where

- *C* is the number of distinct categories that appear in either the ground truth annotations or the predictions;
- *TPi* (true positive) is the number of correctly detected pixels that belong to class i, according to the ground truth segmentation;
- *FPi* (false positive) is the number of pixels that the model incorrectly detects as belonging to class i when, according to the ground truth segmentation, they do not;
- *TNi* (true negative) is the number of pixels correctly detected as not belonging to class i, according to the ground truth segmentation;
- *FNi* (false negative) is the number of pixels that actually belong to class i, according to the ground truth segmentation, but which the system failed to detect.

A *IoU* score of 1 signifies a perfect match between the predicted and ground truth segmentations. However, if a class is absent in both the prediction and the ground truth, it also results in an *IoU* of 1. This highlights the need for careful interpretation, particularly in cases where multiple teeth are missing, as the metric can give a false sense of accuracy in such scenarios.

**Mean Dice**

The Dice coefficient (also known as the Dice Similarity Coefficient (*DSC*) or F1-score) is a widely used metric to evaluate the accuracy of segmentation algorithms [34]. It measures how well the predicted segmentation of an object matches the ground truth, and it ranges from 0 to 1, where 1 indicates perfect agreement between the predicted and ground truth segmentation.

*DSC* is defined as

$$DSC\left(i\right)=\frac{2\;\cdot \;Inter\sec tionArea}{\mathrm{Pr}edictedArea\;+\;GroundTruthArea}=\;\frac{2\;\cdot TPi}{2\;\cdot \;TPi\;+\;FPi\;+\;FNi}$$

The Mean Dice Score (*mDSC*) is the average of the Dice scores across all categories. If there are C categories, the formula is

$$mDSC=\frac{1}{C}\sum_{i=1}^{C}DSC\left(i\right)$$

*mDSC* provides an overall measure of segmentation performance across multiple classes. It is a balanced measure between precision and recall, with values closer to 1 indicating better segmentation quality.

**Mean Precision**

The mean precision measures how many of the predicted positive pixels are actually part of the ground truth [35]. It is defined as

$$m\mathrm{Pr}ecision=\frac{TruePositiveArea}{TruePositiveArea+FalsePositiveArea}=\frac{1}{C}\sum_{i=1}^{C}\frac{TP_{i}}{TP_{i}+FP_{i}}$$

**Mean Recall**

Recall (also known as sensitivity or true positive rate) measures how many of the actual positive pixels were correctly predicted. It focuses on the completeness of the predictions made by the model [11]. The mean recall is defined as

$$mRecall=\frac{TruePositiveArea}{TruePositiveArea\;+\;FalseNegativeArea}=\frac{1}{C}\sum_{i=1}^{C}\frac{TPi}{TPi+FNi}$$

#### 2.3.2. Statistical Analysis

Statistical analysis was performed to evaluate the segmentation performance of ORTHOSEG and to assess potential differences across demographic subgroups. Descriptive statistics, including the mean and standard deviation, were calculated for all performance metrics. Comparisons between male and female patients were conducted using a two-tailed independent samples *t*-test, while differences among age groups were evaluated using ANOVA. A significance level of *p* < 0.05 was adopted for all statistical analyses.

## 3. Results

The model was evaluated on a diverse dataset comprising 50 orthopantomograms, 50 bitewings, and 50 periapical radiographs, totaling 150 images. Segmentation results for all supported radiograph types are presented in Table 5, Table 6, Table 7, Table 8 and Table 9, with an *mDSC* of 0.635 ± 0.222.

Table 6 provides the mean inference time for each type.

Table 7, Table 8 and Table 9 detail the results for orthopantomograms, bitewings, and periapicals, respectively.

The system achieved an *mDSC* of 0.756 ± 0.174 for orthopantomograms with a mean processing time of 19.745 ± 3.625 s, an *mDSC* of 0.705 ± 0.154 for bitewings with a mean processing time of 8.467 ± 0.903 s, and an *mDSC* of 0.445 ± 0.197 for periapicals with a mean processing time of 5.653 ± 0.897 s.

To investigate potential performance variations based on demographic factors, the data was analyzed by sex and age group. Table 10 presents the metrics analyzed by sex, with *p*-values obtained from a two-tailed independent *t*-test.

Table 11 summarizes the results grouped by age, with *p*-values calculated using ANOVA to assess differences among age groups.

**Figure 1:**
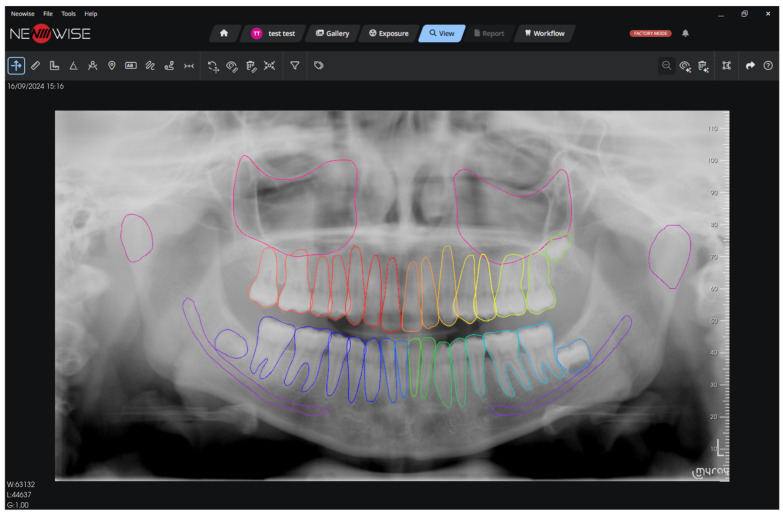
Orthopanoramic segmentation.

**Figure 2:**
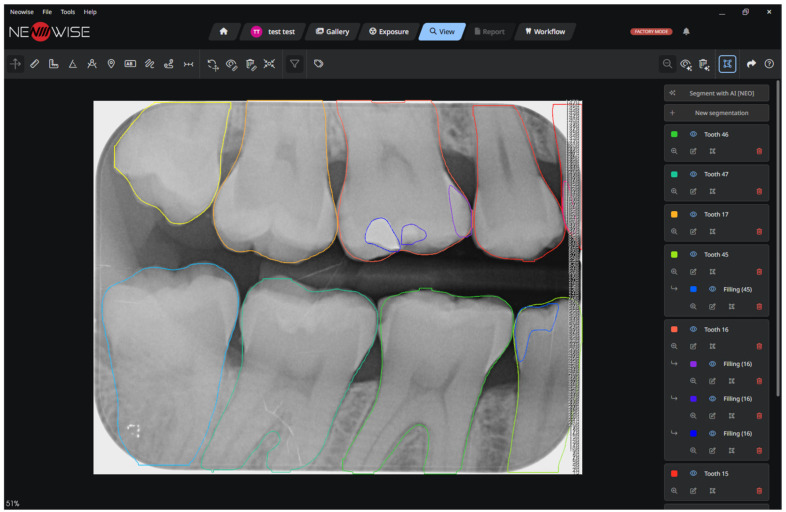
Bitewing segmentation.

**Figure 3:**
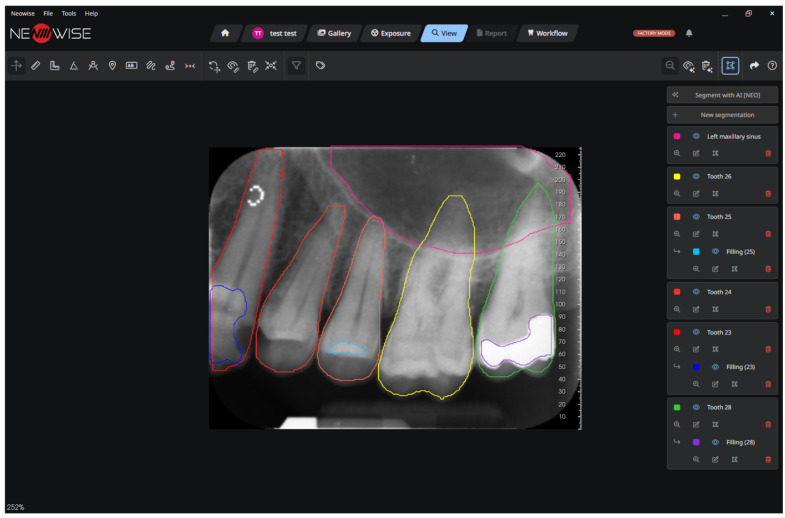
Periapical segmentation.

The analysis revealed no statistically significant differences in performance metrics between sexes. However, a statistically significant difference was observed among age groups. This can be attributed to the increased complexity of radiographs from elderly individuals, which often feature a greater abundance of both pathological and non-pathological elements compared to those of children, adolescents, and adults. Figure 1, Figure 2 and Figure 3 show the output of the ORTHOSEG for the three supported types of radiographs: orthopanoramic, bitewing and periapical, respectively.

## 4. Discussion

A rigorous evaluation of ORTHOSEG was conducted to assess its ability to segment anatomical, pathological, and non-pathological structures from dental radiographs. The study employed a dataset prepared by two experienced clinicians, who followed a dual-review protocol. One clinician performed manual annotations using CVAT, while the second clinician conducted a comprehensive review and quality assurance process. This approach, coupled with stringent checks to address segmentation consistency and image resolution, ensured that the dataset accurately represented real-world clinical scenarios.

ORTHOSEG showed strong performance, particularly for orthopantomograms and bitewings, achieving an *mDSC* of 0.756 ± 0.174 and 0.705 ± 0.154 and an *mIoU* of 0.684 ± 0.172 and 0.652 ± 0.151, respectively. Notably, these results were achieved while identifying and segmenting 70 distinct elements, including anatomical, pathological, and non-pathological structures, an extent of recognition that surpasses many existing systems [8]. This capability highlights ORTHOSEG’s potential to manage complex datasets effectively, ensuring a comprehensive analysis of radiographic images.

When compared to existing state-of-the-art solutions, ORTHOSEG demonstrated promising results. For example, U-Net and Faster R-CNN models have achieved an *mIoU* of 0.501 and an *mDSC* of 0.569 in dental segmentation tasks [5]. ORTHOSEG’s results exceed these benchmarks in terms of the quality and quantity of elements detected, suggesting its capability to deliver competitive performance in dental radiograph analysis while addressing a broader range of identifiable features. The results of the present study are consistent with previous research demonstrating the effectiveness of deep learning models in dental radiograph segmentation. Previous studies have reported Dice coefficients up to 0.569 and *mIoU* values up to 0.501 for periapical radiograph segmentation using U-Net-based architectures [5,15]. Similarly, high segmentation performance has been reported in periapical radiographs, with F1-scores up to 0.88, confirming the potential of deep learning systems for detailed radiographic analysis [22]. In bitewing radiographs, CNN-based segmentation models have achieved F1-scores of approximately 0.84 and diagnostic accuracies up to 0.87, supporting the reliability of artificial intelligence in dental radiographic analysis [9,10]. These findings are consistent with the performance observed for ORTHOSEG, particularly in orthopantomograms and bitewing radiographs. Furthermore, systematic and scoping reviews have emphasized the growing role of convolutional neural networks in improving diagnostic accuracy and supporting clinical decision-making in dentistry [4,11,21].

In addition to its segmentation accuracy, ORTHOSEG achieved efficient computational performance, with average processing times of 19.745 ± 3.625 s for orthopantomograms, 8.467 ± 0.903 s for bitewings, and 5.653 ± 0.897 s for periapical radiographs. These speeds were achieved on hardware commonly available in clinical settings, which suggests that the system could be easily integrated into routine workflows without requiring specialized computational infrastructure.

These results highlight the superior performance of the model on orthopantomograms, while the lower performance on periapical radiographs is attributed to the inherent complexity of these images, particularly in classifying individual teeth.

The lower segmentation performance observed in periapical radiographs may be attributed to several modality-specific factors. Compared to orthopantomograms and bitewings, periapical radiographs provide a more limited field of view and fewer surrounding anatomical reference structures, reducing the contextual information available for accurate segmentation. In addition, periapical radiographs often exhibit greater variability in tooth positioning, angulation, and anatomical overlap, which can increase segmentation complexity. Furthermore, in the present dataset, periapical radiographs included a higher proportion of elderly patients, who more frequently present restorations, implants, and structural alterations, potentially increasing image heterogeneity. These findings suggest that segmentation of periapical radiographs represents a more challenging task for AI systems and highlight the importance of continued optimization and validation.

The system’s segmentation results were consistent across sexes and children and adult age groups, reflecting its robustness and generalizability. However, a slight decrease in accuracy was observed when the system was applied to radiographs of older patients (65+ years), likely due to the increased complexity of their radiographs, which often feature a greater number of overlapping anatomical and pathological elements [36,37,38,39,40,41,42]. These findings underline the system’s adaptability while emphasizing the continued need for clinician oversight in managing complex cases.

By automating routine segmentation tasks, ORTHOSEG can reduce the time required for image interpretation, alleviating the cognitive burden on clinicians and enabling them to focus on evaluating complex cases and making informed clinical decisions.

This study has several limitations that should be acknowledged. First, the dataset used for evaluation was derived exclusively from a European population without differentiating between ethnic groups. Therefore, the findings may not be fully generalizable to patients from other geographic regions that can have different demographic and epidemiological characteristics. Second, dealing with AI systems means dealing with variability. Although the dataset was carefully curated and reviewed by experienced clinicians, it may not capture the full variability encountered in everyday practice, particularly in cases with rare pathologies or unusual anatomical conditions. Finally, the study focused on the system’s performance without analyzing its usability in the Neowise software, its clinical interaction, or its impact on diagnostic workflows.

Future work includes expanding the dataset to cover a broader and more representative patient population and include more imaging devices in order to confirm the system’s generalizability beyond the European sample examined in this study. Future research should focus on expanding the validation dataset to include a broader range of patient demographics and clinical scenarios, further refining the system’s performance and generalizability. This will ensure that ORTHOSEG continues to meet the evolving needs of modern dentistry, cementing its role as an indispensable tool in routine clinical practice.

Moreover, future studies could include prospective clinical trials in which clinicians would use the ORTHOSEG system on new patients, record its outputs in real time, and assess how its segmentation affects diagnostic decisions, reporting times, and workflow efficiency [43,44,45,46,47,48]. Finally, studies on user experience and usability among clinicians will be fundamental to ensure the effective integration of this AI-assisted tool into daily routine dental practice.

## 5. Conclusions

The results of this study confirm that ORTHOSEG meets the performance standards necessary for clinical integration, demonstrating its capability as a reliable and efficient tool for dental radiograph analysis in routine clinical practice. The system’s consistent, high-quality segmentation across various radiographic modalities underscores its potential as a valuable support tool for dental diagnosis and treatment planning. While the system demonstrates robust performance, manual verification by a clinician remains essential, particularly for cases with unique anatomical features or imaging artifacts. This ensures the highest reliability and accuracy in clinical practice. However, the combination of accurate segmentation, efficient processing times, and compatibility with standard clinical hardware highlights ORTHOSEG’s capacity to enhance diagnostic workflows in everyday clinical settings, ultimately contributing to improved patient care.

## Figures and Tables

**Table 1:** Elements detected by the ORTHOSEG system.

Segmented Elements	Segmentation Criteria
Permanent and deciduous teeth (each tooth is labeled according to the FDI notation)	Segmentation should cover the entire tooth structure, from the root tips to the top of the enamel, including all internal components such as the pulp chamber, root canals, and any fillings or caries.
Right (left) mandibular condyle	Segmentation should include the entire right (left) mandibular condyle, covering its cortical and cancellous bone regions.
Right (left) inferior alveolar nerve canal	Segmentation should trace the entire right (left) inferior alveolar nerve canal, from its entrance at the mandibular foramen to its exit at the mental foramen.
Right (left) maxillary sinus	Segmentation should cover the complete left maxillary sinus cavity, outlining its bony boundaries.
Orthodontic band	Segmentation should cover the entire orthodontic band, including all surfaces and attachments to the tooth.
Bracket	Segmentation should include the entire orthodontic bracket.
Crown	Segmentation should cover the complete dental crown, including its outer surface and its core.
Root canal treatment	Segmentation should encompass the entire root canal treatment area, including the filling material from the root apex to the coronal seal.
Implant	Segmentation should cover the full dental implant, from its apical tip to the top of the fixture.
Filling	Segmentation should include the complete filling material.
Endocanal pin (a.k.a. Endodontic post)	Segmentation should encompass the entire post within the root canal.
Cast abutment pin (a.k.a. Metal post and core)	Segmentation should encompass the entire fused metal post and core structure.
Piercings, jewelry, hair clips, or hearing aids	Segmentation should cover the entire object, such as piercings, jewelry, hair clips, or hearing aids, visible within the X-ray field.
Ghosting effect due to piercing, jewelry, hair clips, or hearing aids	Segmentation should include the ghost image artifacts caused by objects such as piercings, jewelry, hair clips, or hearing aids.
Pontic (intermediate element of a prosthetic bridge)	Segmentation should include the entire pontic element.
Bridge	Segmentation should include the entire prosthetic bridge, including the supporting structures and all intermediate elements (pontics).
Internal screw between crown and implant	Segmentation should cover the entire internal screw that connects the crown to the implant fixture.
Caries	Segmentation should cover the entire extent of the carious lesion, including any demineralized or decayed areas of the tooth.
Lesion associated with a tooth	Segmentation should encompass the full extent of any lesion directly associated with a tooth, including periapical, periodontal, or other types of tooth-related pathology.

**Table 2:** Distribution of orthopanoramic radiographs by origin, age and sex.

Population		System Evaluation (Test) Dataset—N	System Evaluation (Test) Dataset—%
Sex	Male	28	56.00%
	Female	22	44.00%
Age	Children (8–12)	6	12.00%
	Adolescents and adults (13–65)	32	64.00%
	Elderly (>65)	12	24.00%

**Table 3:** Distribution of bitewing radiographs by origin, age and sex.

Population		System Evaluation (Test) Dataset—N	System Evaluation (Test) Dataset—%
Sex	Male	25	50.00%
	Female	25	50.00%
Age	Children (8–12)	6	12.00%
	Adolescents and adults (13–65)	27	54.00%
	Elderly (>65)	17	34.00%

**Table 4:** Distribution of periapical radiographs by origin, age and sex.

Population		System Evaluation (Test) Dataset—N	System Evaluation (Test) Dataset—%
Sex	Male	22	47.83%
	Female	24	52.17%
Age	Children (8–12)	5	10.00%
	Adolescents and adults (13–65)	8	16.00%
	Elderly (>65)	37	74.00%

**Table 5:** Evaluation results for all supported radiograph types.

Metric	Mean	Standard Deviation
*mDSC*	0.635	0.222
*mIoU*	0.576	0.214
*mPrecision*	0.661	0.220
*mRecall*	0.628	0.219

**Table 6:** Inference time in seconds (s).

Image Type	Mean	Standard Deviation
Orthopanoramic	19.745	3.625
Bitewing	8.467	0.903
Periapical	5.653	0.897

**Table 7:** Evaluation results for orthopanoramic radiographs.

Metric	Mean	Standard Deviation
*mDSC*	0.756	0.174
*mIoU*	0.684	0.172
*mPrecision*	0.769	0.180
*mRecall*	0.760	0.161

**Table 8:** Evaluation results for segmentation from bitewing radiographs.

Metric	Mean	Standard Deviation
*mDSC*	0.705	0.154
*mIoU*	0.652	0.151
*mPrecision*	0.735	0.159
*mRecall*	0.691	0.148

**Table 9:** Evaluation results for segmentation from periapical radiographs.

Metric	Mean	Standard Deviation
*mDSC*	0.445	0.197
*mIoU*	0.391	0.185
*mPrecision*	0.480	0.197
*mRecall*	0.432	0.195

**Table 10:** Evaluation results grouped by sex.

Metric	Male		Female		*p* Value
	Mean	Standard Deviation	Mean	Standard Deviation	
*mDSC*	0.651	0.242	0.627	0.201	0.504
*mIoU*	0.593	0.232	0.566	0.196	0.444
*mPrecision*	0.676	0.237	0.653	0.203	0.530
*mRecall*	0.645	0.243	0.619	0.194	0.478

**Table 11:** Evaluation results grouped by age.

Metric	Children (7–12)		Adolescents and Adults (13–64)		Elderly (>65)		*p* Value
	Mean	Std	Mean	Std	Mean	Std	
*mDSC*	0.722	0.149	0.727	0.182	0.520	0.222	<0.0001
*mIoU*	0.655	0.146	0.668	0.174	0.462	0.214	<0.0001
*mPrecision*	0.750	0.146	0.751	0.179	0.547	0.223	<0.0001
*mRecall*	0.710	0.150	0.716	0.184	0.517	0.220	<0.0001

## Data Availability

The original contributions presented in this study are included in the article. Further inquiries can be directed to the corresponding authors.

## Abbreviations

The following abbreviations are used in this manuscript:

*DSC*	Dice Similarity Coefficient
*mDSC*	mean Dice Similarity Coefficient
*IoU*	Intersection over Union
*mIoU*	mean Intersection over Union
AI	Artificial Intelligence
CNN	Convolutional Neural Network
GDPR	General Data Protection Regulation
ANOVA	Analysis of Variance
